# Urology in Pediatrics

**DOI:** 10.3390/children9030337

**Published:** 2022-03-02

**Authors:** Giovanni Cobellis

**Affiliations:** Unit of Pediatric Surgery, Salesi Children’s Hospital, Marche Polytechnic University, 60121 Ancona, Italy; giovanni.cobellis@ospedaliriuniti.marche.it

Pediatric urology has been developed by many pediatric urologists and pediatric surgeons from all over the world. Congenital urinary tract malformations are the main field of interest of urology in pediatrics. Obstructive uropathies and vesicoureteral reflux are the most frequent urinary tract congenital anomalies requiring urological consultation. Antenatal and perinatal diagnosis allows early management of these anomalies, before the onset of symptoms, such as urinary tract infections and pyelonephritis. Considering surgical treatments, the most noteworthy advance in pediatric urology has been the development of minimally invasive surgery including endourology, laparoscopy, retroperitoneoscopy and robotic surgery, which have the advantages of reducing postoperative pain, improving the cosmetic results and reducing the time of postoperative hospitalization. The Special Issue “Urology in Pediatrics” mainly focused on the surgical management of urogenital pathology in children, including conservative treatments and minimally invasive and open surgery.

Regarding the conservative management of urinary tract malformations, antibiotic prophylaxis is the basis of treatment of uropathic patients, in order to prevent urinary tract infections and consequent possible renal scarring. Pyelonephritis (PN) is the most serious complication in urophathic patients. Giovanni Parente and colleagues carried out a retrospective study on pyelonephritis (PN) in pediatric uropathic patients. They found that pathogens of PN in uropathic patients, with vesicoureteral reflux (83% Gram-negative bacteria vs. 17% Gram-positive bacteria), are different from those of community-acquired PN (99.1% Gram-negative bacteria vs. 0.9% Gram-positive bacteria), and considering this finding, clinicians should be aware of their peculiar antibiotic susceptibility [1]. Silvia Ceccanti and colleagues reported an original experience in the non-operative treatment of posterior urethral valves in unstable premature babies using a prolonged indwelling urethral catheterization [2].

Endourology is the most minimally invasive approach for treatment of congenital uropathies, such as vesicoureteral reflux and ureterocele. Raffaella Cocomazzi and colleagues performed a retrospective study on the long-term follow-up of the endoscopic treatment for vesicoureteral reflux using two bulking agents, polymethylsiloxane (PDS) and hyaluronic acid/dextranomer (Ha/dx). The reported persistence rate was significantly higher in patients treated with absorbable bulking agents, such as Ha/dx (43%), compared to PDS (25%), and in patients with bladder dysfunctions [3]. Sachit Anand and colleagues performed a systematic review and meta-analysis comparing laser puncture vs. electrosurgical incision techniques for ureterocele decompression. They found a significantly low incidence of de novo VUR and requirement of anti-reflux surgeries in patients treated with the laser puncture technique [4].

Minimally invasive surgery is largely applied in pediatric urology. Laparoscopy, retroperitoneoscopy and, more recently, robotic surgery are the approaches which have been developed in children. Hydronephrosis secondary to uretero-pelvic obstruction is one of the main indications for minimally invasive surgery in children. Anderson–Hynes dismembered pyeloplasty, first described in 1949, remains the gold standard technique. Robotic pyeloplasty seems to be particularly indicated in children more than one year of age, but there is no consensus about the timing of surgical intervention and the appropriate technique for asymptomatic neonates and infants. Mario Lima and colleagues reported a retrospective study comparing patients who underwent an early pyeloplasty, in the first 90 days of life (group 1), with patients who underwent a delayed pyeloplasty, between 3 and 12 months (group 2). They used one-trocar-assisted pyeloplasty (OTAP), which is a video-assisted retroperitoneoscopic technique particularly feasible in small children. They found no significant differences in terms of intraoperative data and early postoperative outcomes, between the two groups. Moreover, they interestingly registered a significant improvement in those patients with an impaired split renal function (SRF) that underwent an early surgical correction, especially in terms of urinary flow, evaluating the hydronephrosis severity score (HSS, which considers the antero-posterior diameter, the level of SRF and the entity of the urinary flow with a MAG-3 renal scan) one year after surgery [5].

Another important topic reported in the Special Issue “Urology in Pediatrics” is surgery of genital acquired pathology. Circumcision is one of the most common urologic procedures performed in children. Laura E. Gilberston and colleagues reported a study on the utilization of an opioid-free anesthetic for circumcision in an ambulatory surgery center. They reported a substantial decrease in in-room and postoperative recovery time with the utilization of this anesthetic approach [6]. Varicocele is a frequent pathology in adolescents, and laparoscopic varicocelectomy has gained growing popularity among pediatric surgeons. Zenon Pogorelic and colleagues performed a randomized controlled trial on the effect of subcutaneous injection at trocar incision sites and intraperitoneal instillation of local anesthetics on postoperative pain after laparoscopic varicocelectomy in adolescents. Compared to the control group, the level of postoperative pain was significantly lower in patients who received local anesthetics intraoperatively [7].

Finally, Edoardo Bindi and colleagues published a case report on continent urinary diversion in children. They reported a girl who was affected by a bladder rhabdomyosarcoma and a girl born with bladder exstrophy and treated at birth. In both patients, a complete cystectomy was performed and a continent neo-bladder was created by detubularized ureterosigmoidostomy using, respectively, Mainz pouch II and Cologne pouch techniques. They concluded that these procedures are safe with good long-term outcomes and, interestingly, are particularly suitable for patients from poor countries because they require no device (bags or catheters) [8].

In conclusion, this Special Issue reports articles regarding different important topics in urology in pediatrics, ranging from the conservative and minimally invasive treatment of urogenital pathologies in children and adolescents, to open approaches for ambulatory and major traditional surgeries in children.
